# Annotations

**Published:** 1905-11-11

**Authors:** 


### ANNOTATIONS.

### Sunday's Milk.

Why should Sunday milk be worse than that sold on Saturday or Monday? There can be no natural reason for it—the cows do their duty without regard to the day of the week; but in Southwark, at least, the milk sold on Sunday is more frequently found to be adulterated than on any other day. Mr. Weatheritt, the inspector for the Food and Drugs Act in that district, has found that Sunday milk is not only worse than that of week-days, but that the badness of it is steadily increasing. Thus in 1903 he found that Sunday milk was worse than that of other days by 1.70 per cent., but in 1904 the percentage had increased to 6.43, and in the present year to 11.30 per cent. Pure milk does not seem to be too plentiful in Southwark at the best of times, for the total average of adulterated milk for these three years was 14.20 per cent. of all the samples taken; but in the same period the Sunday samples showed 20.82 per cent. of adulteration. Moreover, as the day of rest goes on, the milk adulterator seems to become more active. Mr. Weatheritt seems to have pursued his investigations systematically, and the result of them is that he found that of the milk delivered early on Sunday morning in 1905, 17.72 per cent. of samples were adulterated, the late morning milk showed 30 per cent. of adulteration, and the evening milk no less than 40 per cent. of adulteration. The inference is obvious—that the milkman relies on the general cessation of work on Sunday to save him from unpleasant inquiries as to the quality of his milk. The lesson also is obvious—that there is no rest-day for the inspector who wishes to keep the tradesmen in his district up to the mark. It would be interesting to hear the experience of the inspectors in other districts of the Metropolis.

### Alcoholism as a Notifiable Disease.

At an inquest at the Hampstead Coroner's Court on Tuesday into the circumstances surrounding the death of Mr. James Frederick Symonds, who committed suicide by cutting his throat with a piece of glass in a disused nursery, Mr. Mark H. Judge gave evidence which, supported by that of the medical man who attended him, conclusively demonstrated that the deceased was a victim of chronic alcoholism. Mr. Judge, in concluding his evidence, said that the case showed the necessity for treating the disease of alcoholism like infectious diseases, and he appealed to the jury to add a rider to their verdict to that effect. The jury responded to the appeal by recommending that all such cases should be notified to the public health authorities, "whose duty it should be to see that the patient receives proper treatment as in the case of infectious diseases." We appreciate the motives which prompted both the appeal of Mr. Judge and the recommendation of the jury, and we have no doubt whatever that chronic alcoholism ought to be treated as a disease, but not as an infectious disease. In the first place alcoholism is not infectious, and a special Act of Parliament would be required to render it compulsorily notifiable. In the second, what would be the value of notification unless it could be accompanied with power to enforce removal and restraint? This would involve the opening up of many other serious questions, and altogether we feel the proposal must be rejected as quite impracticable. There will always, we fear, be a percentage of suicides due to chronic alcoholism, but we are not prepared, for the sake of the mere possibility of averting an occasional tragedy, to advocate a drastic change in the law which would not only prove to be futile, but might be the cause of far greater evils than it could prevent.

### A New Medical School.

It is with pleasure that we announce the approval by the Board of Management of the Seaman's Hospital Society of a scheme for the establishment of a post-graduation school at Greenwich. We have constantly supported the movement, which had its birth only a few years ago, to provide facilities for post-graduation study. The science of medicine is making such rapid strides that it is necessary for every practitioner who is anxious not to fall behind the times to freshen up his knowledge by attendance at a hospital where clinical teaching is provided. The recognised teaching hospitals have been found not to supply this need. The qualified practitioner, imbued with the more serious aspects of his profession, too often finds an uncongenial companion in the medical student whose buoyant spirits and experience yet require the mellowing influence of time. In addition, the dogmatic teaching which is appropriate for the student is not quite suited to the needs of men who have been already in active practice; so that the setting apart of certain hospitals for post-graduation study has been essential. But the demand has grown very rapidly in recent years, and it is more than probable that "The London School of Clinical Medicine," as it is to be called, at Greenwich, will be found to attract a large number of qualified students without encroaching in the least upon existing institutions whose objects are similar. The Hospital at Greenwich is admirably suited for the purpose of post-graduation study. Moreover, it is intended that the school shall be equipped in the most modern and efficient manner. Reading rooms, library, museum, and well-equipped laboratories will be constructed, and arrangements will be made for providing luncheon and tea to those attending the hospital cliniques. As the patients in the "Dreadnought" Hospital are principally men, special hospitals for women and children will be affiliated for the purposes of affording complete material for instruction. The scheme should appeal as strongly to the public as it does to the profession, for people are beginning to appreciate the fact that their welfare in time of sickness will depend upon the medical skill which is available, and it is to the interest of all to cultivate and encourage such skill by all reasonable means. No better way of attaining this object could offer than by practical support of the new London School of Clinical Medicine.

